# Strategies to improve antimicrobial stewardship in surgery: insights from an ethnographic study

**DOI:** 10.1136/bmjopen-2025-112333

**Published:** 2026-01-23

**Authors:** Hazel Parker, Jo Day, Julia Frost, Rob Bethune, Marianne Hollyman, Kieran Hand, Anu Kajamaa, Karen Mattick

**Affiliations:** 1 Pharmacy Department, Royal Devon University Healthcare NHS Foundation Trust, Exeter, England, UK; 2 Health and Community Sciences, University of Exeter, Exeter, England, UK; 3 Colorectal Surgery, Royal Devon University Healthcare NHS Foundation Trust, Exeter, England, UK; 4 Upper GI Surgery, Somerset NHS Foundation Trust, Taunton, England, UK; 5 Medical Directorate, NHS England, London, England, UK; 6 Faculty of Education, University of Oulu, Oulu, Northern Ostrobothnia, Finland

**Keywords:** SURGERY, Implementation Science, Quality Improvement, QUALITATIVE RESEARCH, Antibiotics, Safety

## Abstract

**Abstract:**

**Background:**

There is an urgent need to improve surgical antimicrobial stewardship (AMS), to enhance individual care and reduce population-level antimicrobial resistance, but it is a complex issue.

**Objectives:**

We aimed to conduct an ethnographic study asking what would work in practice to improve surgical antibiotic prescribing behaviour?

**Methods:**

Adopting a socio-cultural-historical perspective, we undertook ethnographic observations of clinical practice (43.5 hours) and semistructured interviews (n=31) with surgical staff, AMS staff and patients at two English National Health Service hospitals. Interview transcripts and observational fieldnotes were analysed using the Framework Approach. Additionally, we integrated stakeholder engagement throughout to ensure the findings were meaningful.

**Results:**

Our analysis of all fieldnotes (based on 43.5 hours of observation) and interview transcripts (n=31 from interviews with 31 different participants) identified that, while surgical staff were aware of antimicrobial resistance, they seldom considered AMS urgent or important in the acute setting where lack of time and the desire to mitigate perceived risk often prevailed. Other *surgical* issues were perceived to dominate senior decision-makers’ focus, thus perpetuating the status quo. Furthermore, attention to AMS was not always prioritised at the organisational level or by resource-limited AMS teams. Consequently, there was an absence of relationships and tools that foreground AMS. Electronic prescribing systems frequently hindered antimicrobial review and exacerbated patterns of siloed inter-disciplinary working, and feedback on antimicrobial prescribing and patient outcomes was largely absent. To improve AMS, surgical teams wanted sustainable improvements which effectively account for the hierarchical relationships, division of labour, rapid workflow and high staff turnover. Infection experts should better integrate into surgical teams to build relationships and trust, and to proactively contribute to patient care.

**Conclusions:**

We offer data-driven, theoretically informed strategies to support change. Contextually appropriate improvements that address the status and visibility of AMS in surgery will be key. Further research is needed to assess the impact and sustainability of the suggested approaches.

**STRENGTHS AND LIMITATIONS OF THIS STUDY**

- Ethnography is an inductive, iterative methodology which supports the development of rich and in-depth understandings that other approaches may miss.
- We adopted a solutions-orientated stance enabling the formulation of data-driven strategies to support improved surgical antimicrobial stewardship (AMS).
- Our recommendations are based on fieldwork in two National Health Service (NHS) hospitals in Southwest England; however, the detailed reporting enables readers to evaluate the transferability to other contexts; divergent settings may need alternative strategies to bring about change.
- We were unable to speak to executive staff who could have bolstered our understanding about AMS prioritisation at the organisational level.
- Ethnographic findings are interpretive and experientially contingent, meaning that this work represents one version and not a single truth.

## Background

 Antimicrobial resistance (AMR) is a universally recognised patient safety threat, implicated in over 35 000 deaths annually in the UK alone,^1^ and recognised as a national priority.^2^,^4^ Key measures to tackle AMR include infection prevention and control, innovation (eg, development of new antimicrobials and diagnostics) and antimicrobial stewardship (AMS), defined as a coherent set of actions which promote responsible antimicrobial use.^5^ However, despite national action plans,^2 6^ guidelines^7^,^10^ and the work of dedicated antimicrobial stewardship teams (AST),^11^ reducing antimicrobial use in hospitals has been relatively unsuccessful^12^—a challenge that is expected to grow with the ageing and increasingly medically complex population.^13^

Surgical specialities, reliant on effective antimicrobials to treat and prevent infection, are particularly high-volume users (second only to intensive care) and data suggest that prescribing could be safely rationalised in England^14^ and beyond.^15^ Furthermore, research has shown that surgical patients are less likely to be prescribed guideline-compliant therapy compared with medical patients.^16^

It is recognised that AMS interventions tailored to the social and infrastructural context are more likely to lead to improvement,^17^,^19^ and that historically most AMS interventions have not been theoretically informed^20^ or used the most effective behaviour change techniques.^21^ Ergo, there is scope to improve the disconnect between AMS policy and surgical antimicrobial prescribing behaviour (APB). However, while surgical specialities appear to need more AMS input, they have also historically been more difficult to engage.^22^ Qualitative studies have provided insights into the features of surgical teams’ APB,^23^,^36^ highlighting it as distinct from other areas of medicine, and reinforcing the key influence of social and structural mediators.^37^ However, much of the previous research has been undertheorised or has focused on barriers and enablers rather than solutions. Consequently, little is known about how to effectively and sustainably improve surgical AMS. This is important because to increase the impact of interventions, and likelihood that others can replicate them successfully, improvers must be explicit about what they are doing and why, that is, there should be an explicit and well-founded theory of change.^38^

There is an urgent need to improve surgical AMS, to minimise unintended patient harm, but it is complex and poorly understood. Building on our evidence synthesis,^37^ we conducted an ethnographic study to explore what would work in practice to improve surgical antimicrobial prescribing.

## Methods

### Study design

Ethnography is a qualitative methodology that involves observing people in their social environment, drawing on multiple data sources and using the researcher as an instrument.^39 40^ Its inductive, iterative approach can generate rich understandings of social phenomena within healthcare.^41^ We used ethnography to understand complex AMS problems and how to best address them, moving beyond the assumption of a knowledge deficit^42^ and providing insights other methods may miss.^43^ Reporting followed best practice recommendations.^44^

### Theoretical perspective

We adopted a socio-cultural-historical perspective, acknowledging that surgical APB is influenced by many factors including organisational context, past events and workplace relationships.^37^ The multidisciplinary team (MDT) of experienced clinicians and researchers, with subject and methodological expertise, brought complementary interpretive repertoires. Throughout the research, we held regular meetings to collectively question assumptions and to reflect on our own experiences and how they impact interpretation. Additionally, we consulted varied healthcare workers (including surgeons, anaesthetists and pharmacists) and patient representatives to gain their perspectives and ensure our interpretations resonated with their experiences.

### Setting

Two purposively selected National Health Service (NHS) hospitals in Southwest England (table 1) that provide a broad range of surgical services for both elective and emergency patients. As is typical in England, the core surgical teams comprise resident doctors (with a range of levels of experience), physicians’ associates or clinical nurse specialists (at sites 1 and 2, respectively) and consultant surgeons (the most senior surgical grade). Patient care is multidisciplinary, and a range of professions might be involved with antibiotic management including anaesthetists, nurses, perioperative doctors (site 1 only), microbiologists/infectious diseases doctors and pharmacists. For example, anaesthetists typically prescribe and administer surgical antimicrobial prophylaxis (in consultation with the surgeon). However, consultant surgeons have the final say over care decisions because they are clinically responsible for their patients.

**Table 1:** Contextual differences between the two study hospitals

Contextual details	Site 1	Site 2
Type of hospital	University teaching hospital	More rural district general
Number of inpatient beds	886 beds	640 beds
Type of medication prescribing system and medical notes	Single electronic patient record that incorporates the prescribing and medical notes	Standalone electronic prescribing system and paper-based medical notes
Imaging, blood test results and observations	Incorporated within the single electronic patient record (row above)	All in separate electronic systems that do not interface
Daily 8 am ward round format	PTWR: consultant led Upper GI and Colorectal: consultant led Urology: consultant led	PTWR: consultant led Upper GI and Colorectal: registrar (specialist trainee) led (except Mondays in Colorectal which are consultant led) Urology: consultant led
Antimicrobial stewardship provision	Microbiology services (providing diagnostics and advice) Specialist AMS pharmacists Infection management guidelines for common diagnoses available via desktop computers and smartphone apps No regular stewardship ward rounds or MDT meetings in surgery (except weekly infection MDT meetings in vascular and orthopaedics)	Microbiology services (providing diagnostics and advice) Specialist AMS pharmacists Infection management guidelines for common diagnoses available via desktop computers and smartphone apps No regular stewardship ward rounds or MDTs in surgery (except weekly infection MDT meetings in vascular and orthopaedics; and two to three times/week specialist AMS pharmacists identify non-guideline prescribing across the Trust and contribute to patient management as able)

AMS, antimicrobial stewardship; GI, gastrointestinal; MDT, multidisciplinary team; PTWR, post-take ward round in General Surgery (ie, the daily ward round when new admissions are seen by the surgeons).

### Sample

Staff from surgical or allied specialities (eg, microbiology) with surgical antibiotic prescribing experience were eligible for workplace observations and/or interviews. Students (from any healthcare profession) and those under 18 years old were excluded.

Patients were eligible for interview if they were under a surgeon’s care, clinically stable and able to give informed consent and had received systemic antimicrobials during admission. All patients unable to communicate in English, under 18 years old, or considered unsuitable for inclusion by the clinical care team (eg, considered too unwell) were excluded.

### Data collection

Data were collected by one ethnographer (HP) over 15 months (August 2023–October 2024) in a pragmatic, purposive way, guided by the concept of information power.^45^ The ethnographer, an experienced AMS pharmacist (with qualifications including an MPharm, MSc in infection management for pharmacists, a PGDip in prescribing and therapeutics including independent prescribing; and training in qualitative and ethnographic methods from highly experienced teaching teams) working as a clinical-academic and towards her PhD, was explicit about her role and the research aims, ensuring data collection remained overt. Observations were non-participant, involving watching, listening, being present, asking questions and writing fieldnotes.^46^ However, understanding was generated through interactions and embodied experience and was consequently influenced by the ethnographer and communities studied.^47^

#### Workplace observations

Observations were purposively undertaken to study varied surgical situations and staff across general surgery at both sites, focusing on understanding antimicrobial prescribing processes and how they may be improved. Sessions were agreed in advance (via email) with the senior surgeon on shift, and verbal consent was obtained from staff at the start (or as soon as possible), with no incentives offered. Additionally, handover sheets, guidelines and other artefacts were reviewed to provide contextual insights. Unstructured, handwritten fieldnotes and reflections^40^ were typed up on the day of the observations to facilitate rich, accurate recollections (online supplemental information).

#### Interviews

Participants were purposively recruited (via an email from the local surgical stakeholder or face-to-face by the ethnographer during observations) for theoretical representativeness^48^ based on their profession, grade, specialty and workplace to capture a broad range of experiences. Informed consent (written or verbal) was obtained before interviews which were conducted one-to-one in a private setting, either in-person or via videoconference. Discussion loosely followed a topic guide (see online supplemental information) that was developed with reference to AMS and implementation^49^ literature, with stakeholder input and was pilot tested with one resident doctor (who had experience working in both surgery and microbiology). General Data Protection Regulation-compliant audio recordings were professionally transcribed verbatim, anonymised and accuracy checked by HP. Nine predominantly senior participants knew HP professionally (with prior interactions ranging from conversation at network meetings to jointly collaborating on projects) and 12 others met her during observations; these prior relationships helped foster candid conversations. After the interview, participants were offered an optional £30 thank you voucher.

Data collection ran concurrently at both sites, enabling triangulation of data sources and iterative exploration of emerging ideas (eg, exploring observed phenomena with interviewees).

### Analysis

Data were analysed and summarised thematically using the Framework Approach,^50^ involving familiarisation, coding, developing an analytical framework, indexing, charting and interpretation.

The analysis was led by HP in regular discussion with more experienced qualitative researchers (JD, JF and KM)—who supervised and contributed to all stages of the analytic process (eg, through close reading of two initial coded manuscripts, providing reflections and feedback; and through close reading of three early indexed transcripts to review the coding consistency and to support refinement of the analytical framework, interpretive depth, multivoicedness and reflexivity)—and with input from stakeholders and patient representatives. We include inductive and deductive codes, informed by Cultural Historical Activity Theory (CHAT), offering an additional lens for sense-making.^51^ This approach enabled exploration of novel insights, patterns and meaning, while also highlighting theoretically informed aspects (evident during familiarisation), and adding depth to the analysis. CHAT, a socio-cultural theory, is well suited to analysing APB, shifting focus from the individual (as with other behavioural change theories^52^) to encompass their social and cultural surroundings,^53^ which are important.^37^

All interview transcripts and fieldnotes were coded using NVivo 14^54^ and charted in Microsoft Excel.^55^ Subsequently, using various analytic devices,^56^ participants, codes and the relationships between them were considered, including searching for anomalies. Regular researcher dialogue supported reflexivity and refinement until interpretive themes encompassing meaning across the data were agreed. Stakeholder and public/patient involvement and engagement helped ensure the findings are meaningful.

### Patient and public involvement

Patient representatives with first-hand experience of surgical care contributed ideas and feedback throughout the research process, from design to delivery, via formal 90 min stakeholder meetings and at informal one-to-one meetings.

## Results

Fieldwork included 19 observations (43.5 hours) of workplace activity led by 13 different senior decision-makers (table 2) and 31 semistructured interviews (mean duration 53 min; range 8–88 min) with healthcare professionals and patients (table 3). No healthcare workers refused to participate or dropped out from the workplace observations or interviews, and two of five patients offered the opportunity to take part in an interview agreed—non-participants were too tired (n=2) or wanted to focus on other issues (n=1).

**Table 2:** Nature and duration of the ethnographic workplace observations

Observation number	Type of activity observed and specialty*	Location (site)	Duration of observation (hours)†
1	PTWR, general surgery	1	2
2	PTWR, general surgery	2	2.25
3	PTWR, general surgery	1	1
4	PTWR, general surgery	1	1.25
5	PTWR, general surgery	1	1.75
6	Morning ward round, colorectal surgery	1	2.75
7	Morning ward round (and post-ward round jobs with residents and physicians’ associate), colorectal surgery	1	4.75
8	Morbidity and mortality meeting, upper GI and colorectal surgery	1	1
9	Morning handover and ward round, urology	1	3.25
10	Morning handover and ward round, urology	1	2.5
11	Main theatres (including anaesthetic room), colorectal surgery list	1	4.25
12	PTWR, general surgery	1	0.75
13	PTWR, general surgery and colorectal MDT meeting	2	2.75
14	PTWR, general surgery	2	2.25
15	Morning ward round (grand round) and MDT meeting, colorectal surgery	2	2
16	Morning ward round, colorectal	2	2
17	Doctors’ office, colorectal	1	0.5
18	Doctors’ office, colorectal	1	2.5
19	Doctors’ office, colorectal	1	4
			Total=43.5 hours‡

* Ward rounds often started with a patient handover (eg, for the PTWR between night and day staff) and usually culminated in a list run through (plans for each patient reiterated and jobs agreed).

† Rounded down to the nearest 15 min.

‡ Site 1=32.25 hours; site 2=11.25 hours.

GI, gastrointestinal; MDT, multidisciplinary team; PTWR, post-take ward round.

**Table 3:** Characteristics of interview participants by professional role and location

Participants professional roles*†	Number of participants	
	Site 1	Site 2
Surgical team consultants	4	2
Surgical team registrars (specialist trainees)	1	1
Surgical team core trainees	1	1
Surgical team trust grade doctors‡	2	0
Surgical team FY1s	3	5
Surgical team physicians’ associates	1	0
Surgical team advanced nurse practitioners	0	2
Surgical team pharmacists	1	0
Microbiology and/or Infectious diseases consultants	1	1
Antimicrobial stewardship pharmacists	1	1
Perioperative team§ consultant	1	0
Patients	2	0
	Total=18	Total=13

* Surgical team participants worked in a variety or specialities including Upper GI, Colorectal, Urology and Orthopaedics.

† In the UK, the career pathway for a surgeon would typically involve: 2 years of foundation general training (FY1 and FY2); 2 years of core surgical training (CT1 and CT2); 6 or more years of specialist registrar training (ST3-8); possibly additional fellowships; and then gaining a consultant post.

‡ Trust grade doctors are locally employed doctors who are not currently in a training post – their level of experience can vary (both trust grade participants had more than 3 years of experience working as a doctor).

§ The perioperative team is an anaesthetist led service that provide input for surgical patients with complex medical presentations.

Our analysis identified three broad inter-related themes regarding what could be modified to improve surgical APB: (1) ‘status’, who and what is considered important; (2) ‘visibility’ of antimicrobial use and its consequences; and (3) ‘sustainable improvements’ necessitating efficient, persistent strategies for change, elaborated below (see box 1 for illustrative data).

Box 1 **Interpretive themes with examples of illustrative quotations and fieldnotes**

**Theme 1: Status**

[The juniors] feel like it’s the safer thing if they start [antibiotics] and then we can come and say “Actually, you can stop them”. But, obviously, once they’ve been started, it depends on who the senior person is, if they will actually say “No, we should stop them” or whether they say “Oh, well, we’ll wait for the scan” or “Actually, they’re on them now, so we’ll just finish the course”. So, to a certain degree, it’s getting the more junior doctors not to prescribe them if they’re not necessary, but that is difficult because it’s drilled into them so much about the sepsis six … I want to stop them but if it’s kind of borderline and they’ve already been started, then you’d probably go “Oh well, we’ll just carry them on” … [Surgeons] still view it as not particularly important that antibiotics have been started, and they can be stopped or carried on, and … the whole antibiotic resistance thing, although all doctors will know about it, I don’t think it’s viewed as so important in the acute setting… we’re viewed as doing something to someone who’s acutely unwell, and that’s more important than anything else at that point.

Interview, Consultant, GI Surgery (0811)

Unfortunately, most of us are quite defensive in our practice and you feel safer giving someone antibiotics than not. … in the on-call scenario, or the hectic inpatient surgical ward, you do the thing that’s easiest and fastest and least risky, which may not be the … better option … but it’s the fastest and safest option… in a selfish way, [safest] more for the doctor. Because if the patient doesn’t have an infection, it doesn’t really affect them.

Interview, Trust Grade, GI Surgery (1512)

… if you’re either just starting [in surgery] or if you’re more busy, then things like the fact that the patient is still on IV AMG [Amoxicillin, Metronidazole and Gentamicin] and nobody’s reviewed it, just falls by the wayside and nobody notices ‘cos you’ve got so many other things on your jobs list to carry out.

Interview, FY1, GI Surgery (2954)

[Antibiotic review] just needs to be part of the ward round… if the consultant cares about this thing, then the junior cares. …if the consultant’s not regularly checking it, then the juniors are probably not going to be that interested in it…

Interview, FY1, Urology (2091)

The sixth patient was admitted two days ago as an emergency admission with ‘biliary colic/cholecystitis’. The handover list says she was started on ‘oral antibiotics’ although nobody checked the EPMA system. The registrar [leading the ward round] talks to the patient, safety-nets and explains that she can go home this afternoon. The registrar tells the foundation doctor to prescribe five more days oral co-trimoxazole for discharge – meaning the patient will have seven days. Guidelines recommend five days total (no rationale for a prolonged course is given or sought).

Workplace observation, GI surgery ward round

**Theme 2: Visibility**

In some ways [the EPMA system] is great … but it makes some things really, really opaque and difficult to find … one of those things is actually, in some cases, the antimicrobial prescribing.

Interview, Core Trainee, Urology (1496).

[Antibiotic review] is the tricky bit… there aren’t specific [prompts to do this], actually. … if we have been told the patient has been MEWSing high overnight, or if we are thinking about sending them home, these are the main things [that lead to antibiotic review] – either extremes. … [it] is not a universal thing… it is more of a hit and miss thing, I’m afraid.

Interview, Registrar, GI Surgery (0322)

I know a lot over-investigating or over-prescribing of stuff, does, it comes from anecdotal one-off episodes and we’re very bad, in medicine, at acting on our anecdotal experience, rather than consensus information, potentially. Unless the consensus information is clearly going to support us if someone takes us to court for something and we can clearly say that any normal surgeon in this circumstance wouldn’t have done this thing, then it’s fine. But if you think that somebody would call into question your actions, then it makes you much more defensive to do whatever you think the thing is that’s going to protect you more. … so, I think with this very specific thing, I’m going to prescribe [surgical prophylaxis] for seventy-two hours or whatever, I think that’s the motivation behind that.

Interview, Consultant, GI Surgery (2479)

MDT [members] are so stretched that they just keep falling away … so we don’t have that consistency of message or the questions or the double-checking, I think our doctors in training get the pharmacist checking their prescribing electronically, but that doesn’t necessarily lead to better behaviours. … another big challenge is that all our prescribing is electronic now, and whereas before we’d look at every drug chart pretty much on a ward round and now, we don’t look at any. The process [of using the EPMA system] takes too long …. You’ll ask, “Are they on antibiotics?” or “Are they not on antibiotics?”, so you make assumptions, … you don’t automatically have that visual clue of ‘This patient’s been on antibiotics for seven days’, has anyone questioned it? … we’re not looking at the drug charts [EPMA medication record] anymore it’s just too difficult on the ward rounds… ‘Cos the resources aren’t there to make it easy. So, [we’re] not looking at it, [and] we don’t have interaction with colleagues whose role it is to help guide us on those things.

Interview, Consultant, GI Surgery (2892)

**Theme 3: Sustainable improvements**

… the key to improving practice in all the disciplines, is to go face to face with the consultants. … if you start to teach the consultants [at MDT meetings], in a non-threatening environment, then eventually they’ll start telling the juniors. … people don’t like having this stick waved in their face, “You’re wrong and I am right and here’s my stick, I’m going to beat you with, here is the evidence”. That just makes people retreat and entrench themselves in their opinions and say, “I’m not listening to that”. And that’s why, gentle engagement, persistently, by regular meetings and getting people’s trust, is much better than waving the ‘right’ stick. And I think waving the evidence about doesn’t necessarily get you the result you want. You can introduce the evidence later, when you’ve got people’s trust, but if you come in shouting “People, you’re doing it all wrong”, then they’re not going to listen.

Interview, Consultant, Microbiology/Infectious diseases (2194)

It’s selling [change] in the right way, not necessarily for the right reasons of why we’re doing it but selling it in the right way that fits with the people you’re working with, seems to make a big difference …

Interview, Consultant, GI Surgery (2892).

[With infection] MDT meetings … it’s not just you making a decision, in which case they’re [decisions are] on the side of being slightly cautious. … I think people would fall on the side of giving some antibiotics. But if you can go to an MDT [meeting] and talk to colleagues and show them a picture on [the single electronic patient record] and say “Look, this is what the wound looks like, I think it’s OK, CRP is only this, do you think I should give them something?” and if everyone says no, you’ve got that reassurance that you’ve done the right thing and you’ve been backed up by colleagues, so I think that’s important, yeah.

Interview, Consultant, Orthopaedics (0644)

… for me, the kind of constant reminder and a visual reminder of the message is much more beneficial. … if you knew – right, on Wednesdays, the Micro team are coming round with us, we need to be a bit more aware of [AMS] … you’d naturally absorb more knowledge from them, have a better understanding and, in time you’d probably make better decisions with them there. … without a doubt, I think [joint ward rounds are] something that would be very helpful. … it’s difficult when you just have to call someone, sort of intermittently, usually a consultant, and yeah, as a junior, you might not actually have the best grasp of what’s going on with the patient, and you get slightly slammed for not knowing all the information and why you’re calling, ‘cos you’ve just been told by a senior “Oh, speak to Micro about their antibiotics”, well, what exactly am I, you know, being asked to say. … if you can meet regularly and see other teams a lot more, it definitely builds relationships and makes it a lot easier.

Interview, Core Trainee, GI Surgery (2687)

### Status

Status, meaning the level of respect and importance given to something or someone, was key both because of the low relative importance assigned to AMS and because it determined which individuals had the power to influence (and therefore change) practice.

While surgical staff were aware of the concept of AMR, they seldom considered AMS urgent or important when managing the patient in front of them. Against a backdrop of increasing patient complexity, threat of litigation, complaints and diagnostic uncertainty (often requiring scans to make firm diagnoses), participants frequently, although not universally, described preferring to err on the side of overprescribing both as an act of beneficence and to not be caught out personally. Defensive antimicrobial use often provided reassurance, particularly for junior staff, and was perceived as the safest and easiest option, with little consideration of potential downstream negative consequences which did not appear to be especially visible to the staff or patients (Theme 2). There was agreement that teams were quick to start antibiotics, the ‘sepsis six’ messaging has landed (early intravenous antibiotics for sepsis), but slower at reviewing and de-escalation. Once antimicrobials were started, junior staff typically deferred to seniors for plans, with the morning ward round being key for decision-making. However, responsibility for identifying and flagging the need for antimicrobial review remained diffuse and hence easily overlooked, especially if junior staff had recently rotated into the specialty. Furthermore, there was often little appetite for de-escalation (particularly for borderline decisions) as this necessitated a proactive behaviour—a stop and think—which, in the context of uncertainty, competing priorities, diffuse responsibility and perceived (or actual) lack of time, could require more mental bandwidth than was comfortable or easily garnered.

There was widespread recognition that senior leadership and decision-making vis-à-vis antimicrobial prescribing was individualised, impacted by the surgeon’s preferences and comfort with uncertainty, with some consultants described as old-school whereby they preferred intravenous and/or longer antibiotic courses (that others would not use). Hence, variability in senior leadership impacted expectations about how juniors should manage patients, the likelihood of juniors raising questions, the extent of collaboration with infection experts and patient engagement.

Ultimately, antimicrobial review was often reactionary (eg, because the patient was being discharged or blood results markedly changed). If the senior decision-maker did not articulate a clear plan on the ward round, decisions were deferred or specifics left to less experienced doctors to decide, potentially provoking stress, anxiety and a defensive approach (ie, more antibiotics prescribed, for longer). Resident doctors frequently described oral switch/discharge antimicrobials as based on personal preference (senior decision-maker is specific) or arbitrary (if not) and cited usually giving a further 5 or 7 days when left to choose as that seemed reasonable (often longer than guidelines indicated). Other *surgical* issues were perceived to dominate senior decision-makers’ time and focus (eg, assessing for serious complications such as anastomotic leaks or bleeding), perpetuating the status quo, as juniors tended to emulate what they have seen and did not necessarily question it.

In general surgery, attention to AMS was not prioritised by surgeons, organisations (no board-level scrutiny or mandate for improvement), or resource-limited ASTs, who gave precedence to other areas (eg, intensive care and orthopaedics). This apparent lack of urgency and importance attached to general surgical AMS perpetuated the ongoing use of antimicrobials as tools perceived to mitigate patient harm and/or personal blame (ie, they are used to reduce fear, uncertainty and perceived risk) and the acceptance of widespread variation in practice, whereby personal preference can trump evidence-based prescribing and/or timely review is overlooked.

### Visibility

Compounding the problem of status, and because of it, there was an absence of tools, data and relationships to foreground and support AMS in general surgery. Consequently, antimicrobial use and its downstream consequences remained relatively invisible and hence overlooked, creating a false sense of safety (antibiotics perceived as the safest thing) and allowing practice to migrate away from the tenets of AMS.

Owing to staffing shift patterns and competing responsibilities (eg, operating lists), patients and their antimicrobial plans were not necessarily known to team members. Information sharing was essential for continuity and to support decision-making. However, Electronic Prescribing and Medicines Administration (EPMA) systems posed several visibility challenges. On ward rounds, when senior decision-makers were present, it was hard to tell quickly which patients were on antibiotics and whether they needed review. At neither hospital was this information routinely pre-emptively reviewed, partly because of the processes involved (accessing a stand-alone EPMA system when computers were not easily accessible; or navigating through a jumble of orders when the computer was already in use) which can be inhibitory on a fast-paced ward round. Although, notably, no routine workarounds/tools had been established in practice to accommodate the efficient ascertainment of AMS information (unlike, eg, blood results, speaking to the low-ranking status of AMS). Furthermore, participants commented that, when actually sought, antimicrobial prescriptions could be challenging to interpret because of less user-friendly medication displays (compared with iteratively developed drug charts), meaning they were more easily missed, durations were less clear, and they were sometimes inadvertently abruptly stopped. There was no effective mechanism to nudge incorporation of a 48–72-hour antimicrobial review into routine practice.

There were also problems with higher-level data retrieval whereby ASTs were no longer routinely providing specialty-level AMS metrics (eg, guideline compliance), previously cascaded throughout both organisations, owing to complexities in accessing this information after implementing EPMA systems. Prescribers predominantly described having no regular feedback on AMS or infection-related outcomes; therefore, adverse effects remained hidden; there were no data to highlight practice outliers (supporting reflection and/or comparison); and outcomes were largely perceived rather than objective, allowing anecdotal practice, shaped by rituals or single cases, to flourish. Consequently, surgical teams rarely identified antibiotic-associated harm but regularly perceived benefits, perpetuating an unbalanced assessment of the value of antimicrobial use and reinforcing a cautious approach that results in overtreatment. For example, participants described certain colleagues giving 72-hour surgical prophylaxis despite national guidelines recommending a single dose.

Patterns of siloed interdisciplinary working, exacerbated by changes in workflow introduced with EPMA systems, also posed challenges to the prominence of AMS and to inter-disciplinary team relationships. Members of the MDT who could facilitate the realignment of focus onto AMS, to bring it to the forefront of peoples’ minds, were often remote from the surgical environment; did not attend ward rounds (key for decision-making); regularly communicated in ways that were deemed inefficient; and were sometimes unknown to surgical team members, limiting their ability to influence practice. Input from infection specialists was usually reactive and contingent on positive cultures (generally from sites that should be sterile); prolonged intravenous or last-line antimicrobials identified via EPMA systems; or consultation requests from the surgical team (necessitating identification of the need to optimise therapy and prioritisation of help seeking). Furthermore, when advice was sought from infection experts, the task was usually delegated to the most junior resident who may not have the best understanding of the patient’s condition, potentially impacting the consultation outcome. Similarly, both nursing and pharmacy AMS input was perceived as predominantly reactive—for example, if there were issues with intravenous access, or dosing problems and/or drug interactions, respectively—the exception being infrequent interventions from senior practitioners.

### Sustainable improvements

Participants were unanimously open to improving AMS. However, they wanted improvements that are contextually appropriate and there were clear messages around what would make innovations more likely to be acceptable and workable, and therefore sustainable.

Senior surgeons’ buy-in was considered critical to any change; they did not have to lead it but must be onboard with any plan as their teams will act accordingly. Surgeons are clinically responsible for patients and feel accountable for any negative outcomes. Consequently, they will not accept advice they are sceptical about (they tend not to change if they think things are working well) and feedback regarding measures they do not believe in will be ineffective. However, onboarding a collection of autonomous senior surgeons who often have individualised approaches and do not view AMS as an urgent issue was recognised as inherently challenging. It was suggested that, unless change is mandated from above (eg, WHO checklist implementation), which is rare, those seeking to modify practice must take a tailored approach considering individuals’ and departments’ needs and anxieties. Change would need to be presented such that the merits of change and the support align (ie, speak the right language and demonstrate benefits that are appealing to the surgeons). This is not an overnight process; participants consistently recognised presence, continuity, relationship-building and trust as foundational, fostered through face-to-face interaction and coworking (between infection experts and surgeons). MDT meetings and joint ward rounds were valued as they proactively directed attention onto AMS, providing an opportunity for shared decision-making, which functioned as an alternative safety-net. The onus was no longer on an individual to decide, supporting a less defensive approach. Joint rounds and MDT meetings were also felt to provide an ideal forum for education as infection management knowledge could be shared (with the surgical team ideally including decision-makers) supporting improved practice extending beyond purely the cases discussed, as decision-making processes were elaborated and understood, supporting learning. Being discerning of when to give antibiotics (and equally when not to) comes with experience and isn’t something that can just be instilled into people; practitioners need to feel confident that any decision taken keeps them and the patient safe. For this reason, junior staff wanted clear guidelines (explicitly validated by seniors) that they could stand behind in the absence of a senior directing action.

There was also a clear preference for efficient, persistent AMS solutions reflecting the strong, historically established, cultural preference for rapid workflow (which persisted regardless of workload). Participants wanted innovations that saved time and effort (or that at worst were effort-neutral for the surgical team); and that aligned with surgical work patterns, ideally enabling decisions to be addressed when the senior decision-maker was present (otherwise decisions must be checked with senior decision-makers, complicating and potentially diminishing the impact). Owing to the rapid staff turnover, identified as a significant obstacle to practice change, any innovation would have to be continuously communicated and integrated into practice, ideally supported by staff who are embedded and respected within the team and who do not regularly rotate (ie, those who can provide consistency and continuity). Additionally, innovations would have to consider the division of labour (who does what) including tacitly agreed working practices/rules (eg, residents will not prescribe in accordance with guidelines if the senior decision-maker prefers an alternative approach, meaning teaching targeted specifically at residents, eg, FY1s, is unlikely to change practice as a standalone solution). There was broad agreement that increased patient engagement could contribute to enhanced AMS as part of a multifaceted strategy.

In sum, surgical teams wanted improvements that effectively account for the context—including the steep hierarchical relationships, division of labour, rapid workflow and high staff turnover—to make the better thing to do an easier thing to do.

## Discussion

We asked, what would work in practice to improve surgical APB? To our knowledge, this is the first study addressing this question. We identified three inter-related themes that unpack potential improvement targets (status, visibility and sustainable improvements) and inform pragmatic, data-driven strategies for change to strengthen surgical AMS and patient care (see table 4 and the implications for policy, practice and research section).

**Table 4:** Surgical AMS improvement targets with corresponding data-driven strategies for change

AMS improvement targets	Strategies for change
Status	Elevate the urgency and importance of AMS in surgery with enhanced leadership; and explicitly define AMS roles and responsibilities. AMS teams/infection experts should better integrate into surgical specialities, to build relationships and trust, and to proactively participate in AMS.
Visibility	Nudge better AMS with relationships (see row above) and tools that foreground antimicrobial prescription review, for example, via enhanced EPMA system design; and disseminate regular feedback on infection related outcomes (including harm) and the quality of antimicrobial prescribing to support improvement.
Sustainable improvements	Work directly with surgical teams (including senior surgeons) and patients to co-design iterative, long-term improvements which account for the steep hierarchy, division of labour, rapid workflow and staff turnover.

AMS, antimicrobial stewardship; EPMA, Electronic Prescribing and Medicines Administration.

While several studies identify surgical AMS as deprioritised,^37^ our analysis elaborates why, representing a key step towards developing theoretically informed improvements. Status is fundamental, defining who can influence practice and what is considered urgent and important, both linked to hierarchy.^37^ Addressing status, to create urgency around AMS and ensuring AMS leaders have and can enlist others with the requisite power to bring about improvement, will be critical to achieving change.^57^ Clearly defining roles and responsibilities and improving data and feedback may reduce complacency.^57^ However, gaining senior surgeons’ support is paramount and will require leadership and interpersonal effectiveness from infection experts. Until surgical AMS is considered urgent and important, variation in practice will continue, whether intentional (eg, rituals born from a fear^24^) or unintentional (eg, antimicrobial review overlooked). Notably, different mechanisms may be required to address the separate causes of variance.

Our assertion that antimicrobials are used as tools to protect the patient and prescriber (the safest, fastest and least risky choice) is consistent with research in other contexts that frames antimicrobials as gap-filling infrastructure^58 59^ and a means to deflect moral injury.^60^ Surgical teams cannot change their APB until they feel secure that they are not jeopardising their professional reputation or their patients’ health; it would be unreasonable to expect otherwise. Consequently, supportive structures will be required to bring about change, for example, (1) additional senior support for inexperienced juniors; (2) more user-friendly guidelines/policies with explicit senior approval (providing something to stand behind for juniors and consensus information for seniors), that is, an organisational framework that protects the decision-maker and helps reduce fear of negative outcomes which drive action-bias;^37^ and (3) proactive integration of infection experts into surgical care to facilitate shared decision-making,^60^ as an alternative safety-net.

Consistent with other studies,^61^,^63^ our findings indicate that infection experts should work face-to-face with surgical teams, creating a psychologically safe environment and building trusting relationships to effectively influence attitudes and facilitate practice change. Both infection MDT meetings^64^ and AMS ward rounds^65 66^ have been shown to benefit AMS. These initiatives support practice-based learning through which doctors typically acquire their antimicrobial prescribing habits,^61^ and align with central ideas from Bandura’s social learning theory.^67^ Furthermore, antibiotic guidelines^68 69^ and improvements^37^ should be co-designed to support consensus building and a contextually sensitive approach.

Visibility posed several challenges to AMS and will require careful consideration as part of any multifaceted improvement effort. EPMA system vendors must transform interface designs^70^ so antimicrobial prescriptions are clear and conspicuous (as was the case with historic, iteratively designed paper-based drug charts). Additionally, at the local level, application of human factors principles prior to the implementation of EPMA systems^70^ and user-driven refinements could improve usability, reducing barriers to AMS. Our analysis identified the absence of effective tools to reliably prompt a 48–72-hour antimicrobial review. Improving choice architecture^71^ to nudge this into routine practice would support better AMS, for example, thoughtful incorporation of Antibiotic Review Kit principles^72^ into EPMA system design.

The absence of real-time, trustworthy AMS quality and outcome data is problematic on several levels. First, negative consequences remain hidden, perpetuating a view of antimicrobials as the safest thing regardless of other relevant factors (ie, antimicrobial associated harm is not visible and therefore the potential for it is rarely factored into decision-making).^73^ Second, trends in prescribing quality cannot easily be identified and fed back, limiting an assortment of well-recognised behaviour change techniques.^74^ Third, surgeons are understandably more likely to take an entrenched position regarding their APB, to protect their perceived outcomes, if data cannot reassure regarding potential unintended consequences. In short, better data would expose issues for interrogation and make consequences (intended or otherwise) more salient, supporting change.

Surgeons are ready to engage; now is the time for ASTs to provide humble leadership^75^ and support. ASTs must collaborate with surgical teams to co-design and implement long-term, contextually appropriate improvements that account for the interests, values and power relationships that surround them.^76^ Improvements endorsed by senior surgeons, anchored by consistently present, well-respected staff and that require minimal extra effort from the surgical team are more likely to be effective and sustainable.

### Implications for policy, practice and research

AMS improvements must account for contextual factors including status (what and who is considered important); rules, staff continuity and the division of labour (who does what and when); and the rapid workflow including a strong cultural preference for efficiency. Increasing the visibility of antimicrobial use and its consequences will also be key—emphasising the importance of data for action and human factors. Surgical teams will not change their APB unless they feel secure that they are not jeopardising their professional reputation and the health of their patients (eg, when withholding antimicrobials or prescribing shorter courses). We have offered data-driven strategies for change to support improved AMS (table 4). These strategies are intentionally high level, to support transference internationally across a broad range of organisations, and will require tailoring to the local context. Further research should assess the impact and sustainability of these approaches.

### Strengths and limitations

The main strength is the in-depth ethnographic exploration of the context for change, moving beyond the description of barriers to consider how they might be overcome, enabling the formulation of pragmatic AMS improvement strategies. However, ethnography is inherently interpretive, and the results achieved will always be experientially contingent, meaning that researchers will arrive at different interpretations, and this work represents one version and not a single ‘truth’. While our recommendations are based on fieldwork in two NHS hospitals, the detailed reporting enables readers to evaluate the transferability to other contexts;^77^ divergent settings may need alternative strategies to bring about change. Unfortunately, we were unable to speak to executive staff who could have bolstered our understanding regarding AMS prioritisation at the organisational level.

## Conclusions

The lack of urgency and importance assigned to AMS and limited visibility of antimicrobial use and its consequences perpetuate ongoing overuse of antimicrobials as tools to mitigate perceived risk. This study contextualised surgical APB to inform data-driven strategies for improvement. Infection experts and ASTs should proactively build and sustain trusting relationships with surgeons to co-create knowledge and understanding regarding practice change. Improvement strategies must address the social and structural dimensions of the context to make better AMS easier for surgical team to achieve.

## Supplementary material

10.1136/bmjopen-2025-112333 online supplemental file 1

## Data Availability

All data relevant to the study are included in the article or uploaded as supplementary information.
