# Supplementary material for: Strategies to improve antimicrobial stewardship in surgery: insights from an ethnographic study
Source: BMJ Open. 2026 Jan 23;16(1):e112333. doi: 10.1136/bmjopen-2025-112333 (PMC12853513; doi:10.1136/bmjopen-2025-112333)
Supplement: online supplemental file 1 [file bmjopen-16-1-s001.docx]

**Supplementary information and data**

1. Healthcare worker interview topic guide
2. Patient interview topic guide
3. Notes on ethnographic dimensions considered during workplace observations
4. Demographic data for healthcare worker participants
5. Demographic data for patient participants

**ISAPP: HCW interview topic guide**

 INTRODUCTION**:**

- Welcome, introduce self, explain purpose and outline of interview.
- Remind can leave at any time, will record. Any queries?
- Consent.
- Start recording.

THE WAY THINGS ARE

1. **Please can you tell me about your role and how you are involved in prescribing antibiotics?**
2. **What is your experience of the way antibiotics are used in surgery generally and in your area?**

- Can you describe a **typical consultant surgeons/senior decision-makers approach** to antibiotic prescribing?
- **Is it a priority** (if not why not – root cause, if yes what helps with this)?
- Are there any particular **challenges with antibiotic decision-making?**
- When and how prescribed? Is the environment suited to antibiotic decision-making?

1. **Is there clear leadership when it comes to making decisions on antibiotic prescribing?**
   - **Who makes decisions** (does this vary for different types of decision e.g. simple vs complex)?
   - **Is this different at different times?**
2. **Are things different when prescribing prophylaxis vs treatment antibiotics?**
3. **How, if at all, are other members of the MDT involved with antibiotics prescribing in surgery?**

- Who in **MDT**? And in **what ways** – **help or hinder**?
- Is prescribing **delegated**? If yes, when, to who and what types of prescribing? When is it not ok for a junior to make a decision? And how about other HCPs?
- **Is there is scope** for MDT members to take on different roles? And if yes, what?
- **How do other members ultimately impact prescribing decisions** and why is this (e.g. do you follow advice of pharmacist, nurse, microbiologist etc.)?
- Do you and/or the other surgical team members have **confidence in their advice/ability to prescribe?**

1. **To what extent and how are patients involved? What do you think is the impact of this? Do you think they should be?**
2. **How do you think external factors impact antibiotic prescribing (by which I mean national policy and or hospital factors external to your department)?**
3. **Do you get any / much feedback on your antibiotic prescribing?**

- What feedback would be helpful / would be of interest?

THE WAY THINGS COULD BE

1. **Have you identified any areas of especially good practice (here or elsewhere)?**
   - **What makes it** successful? How could we **replicate** this?
   - Would this work in your area (and if not why not)?
   - What would **help/hinder** achieving this? Tools?
   - If you were prioritising one thing, what is the smallest thing we could do to have a positive impact?
2. **How would you like antibiotic decision-making / prescribing to look in surgery?**
   - What would it take for antibiotics to be prescribed ‘perfectly’? (tension for change)

- **Potential solutions** to improve antibiotic prescribing (to reduce unnecessary use)?
- **Why do you think this would be effective** (what is the mechanism)?
- Are there any **potential problems** with this solution?

1. **The literature describes antibiotics as a ‘safety-net’ (i.e. people use them ‘just in case’ / to guard against infection / with a very low threshold) – what are your thoughts on this?**

What do you think could be done to change this ‘safety-net’ approach?

1. **Is there anything else that I haven’t asked that is important / you’d like to add?**

Close:

1. Thanks / next steps in this study / dissemination / voucher.

Post-session: Write up reflection notes electronically / note key points to bring up in the next interview.

RQ: how can we improve surgical antimicrobial prescribing practice?

Objective: to explore what might be workable in practice to modify the status quo so as to improve surgical antimicrobial prescribing.

**ISAPP: PATIENTS interview topic guide**

 Introduction**:**

- Welcome, introduce self, explain purpose and outline of interview.
- Remind can leave at any time, will record. Any queries?
- Consent.
- Start recording.

1. **If you don’t mind, please can you tell me about your surgical admission and how you came to be given antibiotics?**

- How many antibiotics did you have?

1. **Did any healthcare workers talk to you about your antibiotics?**
   1. Do you know/what profession they were?
   2. Did they explain them to you (why you needed them, how long they were for etc.)?
   3. Were you presented with any choices (with regards your antibiotics) e.g. whether you wanted them, what route you’d like them (tablet or into your vein)?
   4. Were any changes to your antibiotics during your stay explained?
2. **Did you go home with antibiotics (and was this explained)?**
3. What were your experiences of taking antibiotics?

- Were there any **problems or difficulties** with your antibiotic therapy or did anything go especially well?
- Did you get side-effects and if yes, who did you tell about them?

1. **Would you like to have been more involved** with your antibiotic therapy (if yes, in what ways)?
2. I am interested in your **experience of the surgical ward round** – please can you tell me about that?
3. **Is there anything else that I haven’t asked that is important** / that you’d like to add?

Close:

1. Thanks / next steps in this study / dissemination / voucher.

Post-session: Write up reflection notes electronically / note key points to bring up in the next interview.

RQ: how can we improve surgical antimicrobial prescribing practice?

Objective: to explore what might be workable in practice to modify the status quo so as to improve surgical antimicrobial prescribing.

**Notes on ethnographic dimensions to consider during workplace observations**

Table describing the type of data to consider collecting when conducting ethnographic observations. Adapted from Charani et al. 2019 <https://doi.org/10.1093/cid/ciy844>

| **Ward Rounds** | **Multidisciplinary Meetings** | **Shadowing individuals** |
| --- | --- | --- |
| - Duration and time of day - People in attendance (roles) - Who lead the ward round - The number of wards visited - The number of patients visited - What was discussed – who lead the discussions, who contributed - What tools were used e.g. electronic prescribing, smartphones, lists - What tasks were identified - Who was responsible for carrying out which tasks - What interactions there were with patients - What interactions there were with other healthcare professionals - What each member of the team did during the ward round i.e. what they contributed or if not contributing what they were doing - Any emotions expressed or felt - Ethnographer contribution, if any, to the activities e.g. pulling curtains - Any disruptions to the activity - Rules – explicit and tacit - Workarounds | - Duration and time of day - Meeting type e.g. Morbidity & Mortality meetings - Who attended (roles) - What was discussed - Who leads the discussion - Who contributed to the discussion - What, if any, data was used or presented - Who presented the data - Any emotions expressed or felt - Rules – explicit and tacit - Workarounds | - Duration and time of day - Type of activity e.g. resident doctor checking bloods in the doctors’ office - Any dialogue between ethnographer and participant - Any disruptions to the activity - The interactions of the healthcare professional with patients and other members of staff - Places visited - Tools used e.g. guidelines, electronic systems, lists - Ethnographer contribution, if any, to the events taking place - Rules – explicit and tacit - Workarounds |

**Notes on capturing workplace observations (Emerson, Fretz and Shaw, 2011)**

Jottings – capture the following:

1. Key components of what you sense are key components of observed scenes, events or interactions.
2. Concrete sensory details about observed scenes and interactions.
3. Avoid characterizing scenes or what people do through generalizations or summaries.
4. Capture detailed aspects of scenes, talk and interaction: short or direct quotes are particularly useful for capturing such detail.
5. Record details of emotional expressions and experiences: note feelings such as anger, sadness, joy, pleasure, disgust, or loneliness as expressed and attended to by those in the setting (but do not presume to know what the ‘reason’ is for an individual’s emotion. Write textured, detailed descriptions of interactions rather than attributing individual motivations.
6. General impressions and feelings, even if you are unsure of their significance at the moment.

Use active rather than passive verbs, sensory rather than evaluative adjectives, and verbatim rather than summarized dialogue.

Because significance can shift/emerge over the course of writing, capture as many minor occurrences, which seem tangential or only vaguely relevant at the time, as possible as these may turn out to provide key insights.

Don’t summarise or use evaluative language – use description, dialogue and characterization.

Description: concrete details; use adjectives and adverbs. Do not let a label stand for a description.

Dialogue: only verbatim in quotation marks. Include gesture, movement, facial expression, tone of voice where possible.

Characterization: telling about a person’s traits is never as effective as *showing* how they act and live. Traits should appear through interactions with others rather than being isolated qualities of individuals.

TIPS: What is going on here? Why are they doing that – explanatory. Local interpretations of how to do things. What is modifiable and what is not?

**Demographic data for healthcare worker participants**

|  | Category |
| --- | --- |
| Gender | 14 Male  15 Female |
| Ethnic group | 7 Asian or Asian British  0 Black, Black British, Caribbean or African  1 Mixed or multiple ethnic groups  18 White  2 Other ethnic group  1 Prefer not to say |
| Age | 8 Aged 20-29 years  11 Aged 30-39 years  4 Aged 40-49 years  4 Aged 50-59 years  2 Aged 60-69 years |
| Location | 16 from site 1  13 from site 2 |

**Demographic data for patient participants**

|  | Category |
| --- | --- |
| Gender | 2 Female |
| Ethnic group | 2 White |
| Age | 1 Aged 60-69 years  1 Aged 80-89 years |
| Speciality | 2 from GI surgery |
| Location | 2 from site 1 |
