# Supplementary material for: Strategies to improve antimicrobial stewardship in surgery: insights from an ethnographic study
Source: BMJ Open. 2026 Jan 23;16(1):e112333. doi: 10.1136/bmjopen-2025-112333 (PMC12853513; doi:10.1136/bmjopen-2025-112333)
Supplement: Supplementary file 2 [file bmjopen-2025-112333.reviewer_comments.pdf]

PEER REVIEW HISTORY

BMJ Open publishes all reviews undertaken for accepted manuscripts. Reviewers are asked to complete a checklist review form and are provided with free text boxes to elaborate on their assessment. These free text comments are reproduced below.

ARTICLE DETAILS

Title (Provisional)

Strategies to improve antimicrobial stewardship in surgery: insights from an ethnographic study

Authors

Parker, Hazel; Day, Jo; Frost, Julia; Bethune, Rob; Hollyman, Marianne; Hand, Kieran; Kajamaa, Anu; Mattick, Karen

---

VERSION 1 - REVIEW

**Reviewer**	**1**
**Name**	**Leone, Marc**
**Affiliation**	**Aix-Marseille Universite**
**Date**	**05-Nov-2025**
**COI**	**None**

The authors nicely showed using an ethnographic designed study the challenge to improve antimicrobial stewardship in surgical patients.

The study addressed a critical point in the perspective of AMR. AMS has been regarded as a major intervention in ID wards, ICU and probably medical wards. Unfortunately, a substantial proportion of antibiotics are used in surgical wards where the concept of reduction of antibiotic use has never been (or merely) considered. This observation seems valid worldwide, questioning the mindset resulting on such failure.

To my view, this ethnographic study just underlined the failure of our processes to rationalise the use of antibiotics in surgical wards, ie in a large part of hospitals. The lack of interest of surgeons for this field is suggested by the authors through the quotes of respondents, as well as the lack of efficiency of the different teams (pharmacist team, ID team, multidisciplinary team...). As we are facing a major healthcare problem, being in the future one of the leading causes of mortality, one can find our "collective" response weak. I would like the authors suggesting proposals, in addition of confirming this situation.

The manuscript is a little bit confined to a British perspective and can be challenging to adhere for non-British readers. In several countries, anaesthetists for instance play a role in

the perioperative medicine, and can interfere between the patients, the surgeon, and the ID experts. Due to their medical training, they are often more prone to perceive the benefits and harms associated with antibiotic use (and overuse).

The non compliance to guidelines, the lack of specific training, and the paucity of expertise (decision led by juniors) is surprising through the manuscript; the more surprising is the kind of resilience of authors facing this situation. It seems that every deviation was admitted because they are surgeons. In the hospital culture, it is probably something we have to work on. Thus such a paper cannot only see the damage without suggesting solutions.

On a formal plan, this type of paper is pleasant to read, but it is too long, and the message would deserve to be condensed with "quotes" from respondents in a separate table or supplemental file (perhaps keep only the most significant). Once again, the journal have an international readership, thus the authors are encouraged to consider that all not readers are aware about the UK hospital operation, An illustration would be helpful.

**Reviewer**	**2**
**Name**	**thursky, karin**
**Affiliation**	**Melbourne Health, Infectious Diseases**
**Date**	**17-Nov-2025**
**COI**	**None**

This study was intended to gain insights into surgical antimicrobial prescribing. It is unclear as to why the researchers have not addressed antimicrobials prescribed before and during surgery. The study seems to have focused entirely on ward-based antimicrobial use.

This is a major limitation to this study, and also a lost opportunity to understand decision-making pre and intraoperatively. The key findings are also not particularly novel concerning hierarchy and lack of prioritisation around antibiotics.

The data around the artefacts used (paper, EMRs) is very interesting and relevant, and in particular, the problems described around the visualisation of antimicrobial prescribing history. This provides an opportunity to discuss the role of human factors and usability in the design of AMS interventions.

In addition, there is an entire group of clinicians who actively prescribe the antimicrobials at surgery, and who were not included during this ethnographic study - anaesthetists. There was only 1 episode of theatre observation, limiting potentially very interesting observations in the immediate postoperative period regarding charting and planning for antimicrobials.

The single ethnographer was known to several of the senior participants. While this would have assisted with rapport and trust, there could have also been risks and biases introduced into the data. The limitations of this should be addressed in the discussion.

Antimicrobial stewardship also includes policies, procedures, audit and feedback, and surveillance. While not the focus of this research program, there could have been an opportunity to explore some of these elements with the participants.

The manuscript itself is well written; however, the style verges on being too casual and less scientific in style.

VERSION 1 - AUTHOR RESPONSE

<table>
<tr><th colspan="3">Reviewer 1: Prof. Marc Leone, Aix-Marseille Universite, Assistance Publique Hopitaux de Marseille.
Comments to the Authors:</th></tr>
<tr><td>12.</td><td>The authors nicely showed using an ethnographic designed study the challenge to improve antimicrobial stewardship in surgical patients. The study addressed a critical point in the perspective of AMR. AMS has been regarded as a major intervention in ID wards, ICU and probably medical wards. Unfortunately, a substantial proportion of antibiotics are used in surgical wards where the concept of reduction of antibiotic use has never been (or merely) considered. This observation seems valid worldwide, questioning the mindset resulting on such failure. To my view, this ethnographic study just underlined the failure of our processes to rationalise the use of antibiotics in surgical wards, i.e. in a large part of hospitals. The lack of interest of surgeons for this field is suggested by the authors through the quotes of respondents, as well as the lack of efficiency of the different teams (pharmacist team, ID team, multidisciplinary team...). As we are facing a major</td><td>Thank you for your comments. Surgical antimicrobial stewardship is a healthcare topic of urgent international importance, requiring effective and sustainable improvement, and we are pleased to have conveyed this message.

With regards to suggesting proposals for change, we very much sought to take a solutions focus approach and regret that this has not come through clearly. Our three overarching themes speak to the solutions/improvement targets (status, visibility and sustainable solutions) and explicate antimicrobial prescribing practice to inform change. The discussion also has proposals for how things could change threaded throughout, for example:
Regarding the use of antimicrobials as tools: ‘supportive structures will be required to bring about change, e.g. (1) additional senior support for inexperienced juniors; (2) more user-friendly guidelines/policies with explicit senior approval (providing something to stand behind for juniors and consensus information for seniors) i.e. an organisational framework that protects the decision-maker and helps reduce fear of negative outcomes which drive action-bias; and (3) proactive integration of infection experts into surgical care to facilitate shared decision-making, as an alternative safety-net’ [line 447-453].
Regarding collaboration between Surgeons and ASTs: ‘our findings indicate that infection experts should work face-to-face with surgical teams, creating a psychologically-safe environment and building trusting relationships to effectively influence attitudes and facilitate practice change…… Further, antibiotic guidelines and improvements should be co-designed to support consensus building and a contextually-sensitive approach’ [line 455-462].</td></tr>
</table>

	healthcare problem, being in the future one of the leading causes of mortality, one can find our "collective" response weak. I would like the authors suggesting proposals, in addition of confirming this situation.	However, to place more emphasis on our solutions focus, we have moved Table 5 (the table that lists improvement targets with corresponding data-driven strategies for change) to the beginning of our discussion [line 427-429]. We have also renamed the column titles with more suitable titles to reflect the solutions focus (the column title 'Problem' has been changed to 'AMS improvement targets'; and the column title 'change strategies' has been changed to 'Strategies for change'). The strategies provided in Table 5 are intentionally high-level to support transference internationally across a broad range of organisations (and we have now explained this in the implications for policy and practice section) [line 498-501].
13.	The manuscript is a little bit confined to a British perspective and can be challenging to adhere for non-British readers. In several countries, anaesthetists for instance play a role in the perioperative medicine, and can interfere between the patients, the surgeon, and the ID experts. Due to their medical training, they are often more prone to perceive the benefits and harms associated with antibiotic use (and overuse).	Thank you. We have elaborated on surgical care processes at our settings in the methods section to clarify additional relevant contextual information and to make the manuscript clearer/easier for diverse readers to follow [line 129-138].<br><br>Similar to other countries, one of our sites had an anaesthetist led peri-operative service and we have now made this clear (one of the consultants from this team was interviewed, see Table 3) [line 129-138 and line 252-253]. We agree that the peri-operative teams tend to be more engaged with medication and antibiotic issues and do not view this as in conflict with our strategies for change. In the English context, the surgeon is clinically responsible for their patients and therefore they have the final say regarding care decisions – hence senior surgical buy-in is critical for change. We also recognise that, with the surgeon's agreement, other professions can play important roles to contribute to AMS hence recommending improvements account for the hierarchy/rules and division of labour. We understand that solutions may be different elsewhere (particularly where processes happen very differently to our settings) and have added to the limitations to reflect this (also see authors' response to reviewer comment four).
14.	The non-compliance to guidelines, the lack of specific training, and the paucity of expertise (decision led by juniors) is surprising through the manuscript; the more surprising is the kind of resilience of authors facing this situation. It seems that every deviation was admitted because they are surgeons. In the hospital	As above, we sought to take a solutions focus and have adjusted the manuscript to bring this into sharper focus (please see authors' response to reviewer comment number 12 above).

	culture, it is probably something we have to work on. Thus such a paper cannot only see the damage without suggesting solutions.	
15.	On a formal plan, this type of paper is pleasant to read, but it is too long, and the message would deserve to be condensed with "quotes" from respondents in a separate table or supplemental file (perhaps keep only the most significant).	Thank you. The quotes are presented in a table however we recognise they are lengthy. In response to your feedback, we have reduced the number of quotes presented (from 16 to 13) and in addition we have significantly reduced the size of several of the remaining quotes. As a result, the text in Table 4 (containing the illustrative quotes and fieldnotes) has now been reduced by one third [line 417].
16.	Once again, the journal have an international readership, thus the authors are encouraged to consider that all not readers are aware about the UK hospital operation, an illustration would be helpful.	Thank you. We have made some changes as per our response to your comment above (please see authors' response to reviewer comment number 13).
**Reviewer 2: Prof. Karin Thursky, Melbourne Health, Peter MacCallum Cancer Institute Comments to the Authors:**		
17.	This study was intended to gain insights into surgical antimicrobial prescribing. It is unclear as to why the researchers have not addressed antimicrobials prescribed before and during surgery. The study seems to have focused entirely on ward-based antimicrobial use.<br>This is a major limitation to this study, and also a lost opportunity to understand decision-making pre and intraoperatively. The key findings are also not particularly novel concerning hierarchy and lack of prioritisation around antibiotics.	Thank you for your comments.<br><br>This study was intended to explore what would work in practice to improve surgical antimicrobial prescribing. While we did focus extensively on ward based antimicrobial use (as this is where much hospital surgical antimicrobial use/prescribing occurs; and stakeholder engagement flagged ward based prescribing as more problematic) we did consider antimicrobial use before, during and after surgery – for example when conducting theatre observation (see manuscript, table 2, observation 11); and during the semi-structured interviews (see supplementary material, e.g. topic guide questions 1-3 encouraged participants to talk about antimicrobial use at any point/stage during the surgical pathway; and question 4 specifically asked for information about surgical prophylaxis). As such, the themes and data-driven strategies for improvement are highly relevant to surgical antimicrobial prescribing across the patient pathway (i.e. the importance of status, visibility and sustainable solutions hold as relevant to both surgical prophylaxis and treatment courses of antibiotics).<br><br>We agree that hierarchy and the lack of prioritisation of antimicrobial stewardship have been highlighted previously (in terms of being important contextual aspects) therefore it

		is fitting that strategies for improvement address these important and challenging aspects (alongside other aspects e.g. visibility).<br><br>With regards our contribution, this research was novel in a number of ways:<br>• We took a solutions-oriented approach. The themes explicate surgical antimicrobial prescribing behaviour providing explanatory nuance to build understanding; and uncovering data-driven strategies for improvement to move the field forward (i.e. we explicitly link important contextual aspects to data-driven strategies to support change).<br>• We provided a novel way to conceptualise surgical antimicrobial use through the assertion that antimicrobials have become tools to protect the patient and prescriber thus necessitating the introduction of alternative supportive mechanisms/tools if things are to change.<br>• We highlight aspects that will be fundamental to sustainable change e.g. the importance of relationships (participants consistently recognised presence, continuity, relationship-building, and trust as foundational, fostered through face-to-face interaction and co-working) and humble leadership.<br>• We highlighted important characteristics to consider when developing improvements – such as status (what and who is considered important), rules, staff continuity, the division of labour (who does what and when) and the rapid workflow including a strong cultural preference for efficiency – suggesting that antimicrobial stewardship needs to be made easier for the surgical team to achieve.<br>• We also draw attention to AMS visibility and the importance of artefacts.<br>In sum, we believe this manuscript offers new ways to think about the intractable problem of surgical antimicrobial stewardship and have taken on board the reviewer feedback to bring our solutions focus into sharper view (please see authors' response to reviewer comment number 12).
18.	The data around the artefacts used (paper, EMRs) is very interesting and relevant, and in particular, the problems described around the visualisation of antimicrobial prescribing history. This provides an opportunity to discuss the	Thank you. We agree and have suggest that the application of human factors principles prior to the implementation of EPMA systems and user-driven refinements could improve usability, reducing barriers to AMS [line 453]. We have now also reiterated the importance of data for action and human factors in the implications for policy, practice and research section [line 495-496].

	role of human factors and usability in the design of AMS interventions.	
19.	In addition, there is an entire group of clinicians who actively prescribe the antimicrobials at surgery, and who were not included during this ethnographic study - anaesthetists. There was only 1 episode of theatre observation, limiting potentially very interesting observations in the immediate postoperative period regarding charting and planning for antimicrobials.	Thank you for this observation. While we agree that additional time spent with anaesthetists may have contributed additional valuable insights[3] we wish to highlight that anaesthetists were included in this study. As you note, there was one long theatre observation session during which the ethnographer was able to observe anaesthetists working and to have wide ranging conversations with them regarding their role, practice and influence – we have added 'anaesthetic room' to Table 2 to signal this [line 238]. The semi-structured interviews also enabled discussions regarding anaesthetists' roles and their contribution to antimicrobial stewardship and decision-making; and the peri-operative medicine service at one site is anaesthetist led – we have added to the manuscript to make this clear [line 252]. Further, we have engaged anaesthetic stakeholders throughout the research programme and as such representatives from the profession have contributed to our understanding. To make it clear that anaesthetists have been involved in stakeholder engagement we have added the professions of the stakeholders that we have consulted to the manuscript [line 124-125].<br><br>Our sampling strategy was purposive and our decision not to spend additional time with anaesthetists reflects the time available and our understanding that, in the UK context, surgeons make the final decision regarding surgical prophylaxis consistent with elsewhere[7 8] (anaesthetists can make recommendations however the surgeon has clinical responsibility for the patient and will ultimately decide).<br><br>As mentioned above (please see authors' response to reviewer comment number 17), the data-driven strategies for improvement are relevant to surgical antimicrobial prescribing across the patient pathway including to surgical prophylaxis.
20.	The single ethnographer was known to several of the senior participants. While this would have assisted with rapport and trust, there could have also been risks and biases introduced into the data. The limitations of this should be addressed in the discussion.	Ethnography is a qualitative methodology that involves observing people in their social environment, drawing on multiple data sources and using the researcher as an instrument [4 5] [line 111-112]. The process is inherently subjective and we agree that our data were influenced by both the communities studied and by the ethnographer [line 163-164].<br><br>In-keeping with our socio-cultural-historical perspective we sought to include many voices in the study (reflecting the importance placed on multi-voicedness[9]). To this end, we

		interviewed a broad range of healthcare workers – some previously known to the ethnographer (to varying degrees) and some not – with a range of professions, specialities, roles and levels of experience from two different hospitals; and observed an assortment of surgical workplace activities relevant to antimicrobial prescribing behaviour. The process was iterative and allowed for the exploration of emerging ideas with participants [line 190-191]. Further, throughout the research process we involved patient representatives and healthcare worker stakeholders; and engaged in regular researcher team meetings to support reflexivity and rigour (e.g. by collectively questioning assumptions and reflecting on our own experiences and how they might impact our interpretation) [line 119-126].<br><br>To acknowledge the interpretive nature of ethnography, we have added to the strengths and limitations section: *'…ethnography is inherently interpretive and the results achieved will always be experientially contingent, meaning that researchers will arrive at different interpretations, and this work represents one version and not a single 'truth'.'* [line 509-512].<br><br>We have also elaborated further on the professional relationship between the ethnographer and the participants: *(…with prior interactions ranging from conversation at network meetings to jointly collaborating on projects)* [line185-186].
21.	Antimicrobial stewardship also includes policies, procedures, audit and feedback, and surveillance. While not the focus of this research program, there could have been an opportunity to explore some of these elements with the participants.	We agree that these are important elements of antimicrobial stewardship and as such were mindful of them during the observations (the ethnographer is an experienced antimicrobial stewardship pharmacist and had access to guidelines and other relevant artefacts [line 157 and line 171]). Further, we did explore these elements with the interview participants (while the interview guide may not explicitly list a question on each element, the interviews were flexible in format [line 180] and these elements were discussed as they are highly relevant to antimicrobial prescribing behaviour. For example, when focusing on question 8 'Do you get much feedback on your antibiotic prescribing?' the ethnographer would probe regarding audit and feedback, surveillance/outcome data, adhoc feedback, key performance indicators etc. Learning from exploration of these elements with participants has contributed to our overarching themes e.g. visibility where we identify that the teams are receiving very limited feedback (very limited surveillance and/or audit data and feedback) and that better data would expose issues for interrogation and make consequences (intended or otherwise) more salient, to support change; and sustainable improvements where we

		identify for example that junior staff wanted clear guidelines (explicitly validated by seniors) that they could stand behind in the absence of a senior directing action.
22.	The manuscript itself is well written; however, the style verges on being too casual and less scientific in style.	Thank you. We acknowledge that the manuscript departs from a traditional scientific style. This was an intentional choice[10] as a less formal, first-person academic voice aligns with interpretivist traditions in the social sciences and allows us to be transparent about our role in the research process[11]. In addition, a more flexible prose style supports the use of storytelling, thick description, and reflexive analysis which are hallmarks of strong ethnographic and qualitative scholarship.

References

1. Gertner AK, Franklin J, Roth I, et al. A scoping review of the use of ethnographic approaches in implementation research and recommendations for reporting. *Implement Res Pract* 2021;2 doi: 10.1177/2633489521992743 [published Online First: 2021/06/01]
2. Varpio L, Ajjawi R, Monrouxe LV, et al. Shedding the cobra effect: problematising thematic emergence, triangulation, saturation and member checking. *Medical Education* 2017;51(1):40-50. doi: 10.1111/medu.13124
3. Braun V, Clarke V. How do you solve a problem like COREQ? A critique of Tong et al.'s (2007) Consolidated Criteria for Reporting Qualitative Research. *Methods in Psychology* 2024;11 doi: 10.1016/j.metip.2024.100155
4. Emerson R, Fretz R, Shaw L. Writing Ethnogaphic Fieldnotes. 2nd ed: The University of Chicago Press 2011.
5. Hammersley M, Atkinson P. Ethnography Principles and Practice. 4th ed. London and New York: Routledge 2019.
6. Malterud K, Siersma VD, Guassora AD. Sample Size in Qualitative Interview Studies. *Qualitative Health Research* 2016;26(13):1753-60. doi: 10.1177/1049732315617444
7. Broom JK, Broom AF, Kirby ER, et al. How do professional relationships influence surgical antibiotic prophylaxis decision making? A qualitative study. *Am J Infect Control* 2018a;46(3):311-15. doi: 10.1016/j.ajic.2017.09.004 [published Online First: 2017/11/11]
8. Ierano C, Thursky K, Peel T, et al. Influences on surgical antimicrobial prophylaxis decision making by surgical craft groups, anaesthetists, pharmacists and nurses in public and private hospitals. *PLoS ONE [Electronic Resource]* 2019;14(11):e0225011. doi: https://dx.doi.org/10.1371/journal.pone.0225011
9. Engeström Y, Sannino A. Studies of expansive learning: Foundations, findings and future challenges. *Educational Research Review* 2010;5(1):1-24. doi: 10.1016/j.edurev.2009.12.002
10. Lingard L, Watling C. From Silent to Audible Voice: Adjusting Register, Stance & Engagement in Your Writing. Story, Not Study: 30 Brief Lessons to Inspire Health Researchers as Writers2021:155-63.
11. Webb C. The use of the first person in academic writing: objectivity, language and gatekeeping. *J Adv Nurs* 1992;17(6):747-52. doi: 10.1111/j.1365-2648.1992.tb01974.x

VERSION 2 - REVIEW

Reviewer 2

Name thursky, karin

Affiliation Melbourne Health, Infectious Diseases

Date 26-Dec-2025

COI

Thank you for your considered responses to the reviewers questions.

These have been answered adequately.

One minor comment is that you have not mentioned the role of nurses - these are critical roles in surgical AMS on the wards. (see Esmita Charani papers)
